# Psychological Factors in Hypothyroidism Subgroups: Childhood Trauma, Alexithymia and Perseverative Thinking

**DOI:** 10.1192/j.eurpsy.2026.12017

**Published:** 2026-07-31

**Authors:** M. Özyıldırım, E. E. Şahinbaş, F. B. Parlakkaya, A. Kurtulmuş, Z. Çelik

**Affiliations:** Istanbul Medeniyet University, istanbul, Türkiye

## Abstract

**Introduction:**

Hypothyroidism is a heterogeneous disorder shaped by biopsychosocial influences. Childhood trauma contributes to hypothalamic-pituitary-adrenal (HPA) axis dysregulation, increasing vulnerability to immune-related diseases (Heim et al.,2008). Alexithymia and perseverative negative thinking are linked to impaired emotion regulation under stress, with psychiatric and immunological consequences. Studying these factors in autoimmune and non-autoimmune hypothyroidism may clarify psychoneuroimmunological mechanisms.

**Objectives:**

This study examined associations between childhood trauma, alexithymia, and perseverative thinking in autoimmune and non-autoimmune hypothyroidism. It further assessed how these psychological dimensions relate to autoimmunity and thyroid pathology, and whether they differ by disease subtype. Findings are expected to highlight the role of affective disturbances in autoimmunity and inform integrative management.

**Methods:**

This observational study is being conducted at the Endocrinology Outpatient Clinic of Prof. Dr. Süleyman Yalçın City Hospital with approval from the Istanbul Medeniyet University Ethics Committee. A total of 114 participants have been recruited between July 2025 and January 2026: autoimmune hypothyroidism (n=54), non-autoimmune hypothyroidism (n=24), and healthy controls (n=36). Participants completed the Beck Depression Inventory, Beck Anxiety Inventory, Perseverative Thinking Questionnaire, Childhood Trauma Questionnaire (CTQ-28), and Toronto Alexithymia Scale (TAS-20). Data were analyzed in SPSS; significance was set at p<0.05. This report presents preliminary results.

**Results:**

After adjusting for age, gender, comorbidities, and psychiatric history, significant differences emerged only in Beck Anxiety scores (F=3.899, p=0.023). Post-hoc tests showed higher anxiety in the thyroidectomy group versus controls (p=0.019). No group differences were found for depression, perseverative thinking, or alexithymia (all p>0.05). CTQ scores did not differ across groups (p>0.05).

**Conclusions:**

Hypothyroidism patients, especially those who underwent thyroidectomy, appear more vulnerable to anxiety. Depression, alexithymia, and perseverative thinking did not vary by disease subtype, suggesting closer links with psychiatric history than thyroid pathology. These findings underscore the need for psychiatric evaluation—particularly anxiety and trauma screening—in the holistic care of hypothyroidism.

**Disclosure of Interest:**

None Declared

